# Matched pair analysis of the accuracy and outcome of navigated screw fixation of the posterior pelvic ring

**DOI:** 10.1007/s00590-025-04394-2

**Published:** 2025-07-01

**Authors:** Felix Karl-Ludwig Klingebiel, Octavia Emilia Sophie Klee, Anhua Long, Henrik Teuber, Michel Teuben, Sascha Halvachizadeh, Jakob Hax, Till Berk, Jochen von Spiczak, Christian Hübner, Adam Starr, Ladislav Mica, Valentin Neuhaus, Hans-Christoph Pape, Yannik Kalbas, Roman Pfeifer

**Affiliations:** 1 Department of Trauma Surgery, University of Zurich, University Hospital of Zurich, Zurich, Switzerland; 2 Harald-Tscherne Laboratory for Orthopaedic and Trauma Research, University Hospital Zurich, University of Zurich, Zurich, Switzerland; 3 Department of Urology, University Hospital of Zurich, Zurich, Switzerland; 4 Department of Orthopaedics, Beijing Luhe Hospital Affiliated to Capital Medical University, Beijing, China; 5 Department of Knee and Hip Surgery, Schulthess-Klinik, Zurich, Switzerland; 6 Harald-Tscherne Laboratory for Orthopaedic and Trauma Research, University Hospital Zurich, University of Zurich, Zurich, Switzerland; 7 Department of Trauma and Reconstructive Surgery, University Hospital RWTH Aachen, Universitätsklinikum Aachen, Aachen, Germany; 8 Diagnostic and interventional Radiology, University Zurich, University Hospital of Zurich, Zurich, Switzerland; 9 Department of Orthopaedic Surgery, The University of Texas Southwestern Medical Center, Dallas, United States

**Keywords:** 3D Navigation, Pelvis surgery, Sacroiliac screw

## Abstract

**Purpose:**

Navigated sacroiliac screw fixation of the posterior pelvic ring has been introduced, providing the surgeon with an improved three-dimensional orientation of the anatomy. The primary aim of this study was to evaluate the influence of navigation on the surgical outcome. The secondary aim was to evaluate the relevance of sacral dysmorphism.

**Methods:**

A retrospective cohort study of patients from 2014 to 2022 admitted with acute traumatic pelvic ring injuries was performed. Patients aged ≥ 16 years treated electively with posterior pelvic ring screw fixation and at least 12 months follow up and informed consent were included. Patients were stratified according to treatment strategy (NAV: Navigated screws vs. CONV: Conventional screws) and compared regarding implant-related complications. Patients were automatically matched 1:1 according to age, gender and fracture stability.

**Results:**

A total of 208 patients were included (NAV: n = 66, CONV: n = 142). After matching, 132 patients were finally included with 66 patients in each. Transsacral screws were used more often in the navigated group (69.7%; OR = 5.58, p < 0.0001). There were no significant differences regarding hardware complications. However, no malpositioning or foraminal breaching occurred in the navigated group. A back-analysis of the unmatched group with elevated power revealed that malpositioning rate in the navigated group was significantly lower (p = 0.033). The presence of sacral dysmorphism did not affect malpositioning rates yet those patients were less likely to receive a transsacral screw instrumentation.

**Conclusion:**

Navigated screw fixation of the posterior pelvic ring resulted in optimal accuracy of screw placement in trauma patients. Sacral dysmorphism scores might support surgical decision-making regarding the choice of screws. Especially patients with poor bone quality might benefit from transsacral screws, which can be introduced more safely using navigation.

**Supplementary Information:**

The online version contains supplementary material available at 10.1007/s00590-025-04394-2.

## Introduction

Current technological advances have enabled increased use of three dimensional navigation systems in spine and pelvic surgery, to enhanced intraoperative orientation and to improved implant placement and fracture fixation (1, 2). Insertion of sacroiliac screws in posterior pelvic ring injuries has benefitted from this technology, but navigated screw placement remains less common than conventional percutaneous instrumentation (3) done with fluoroscopic guidance (4, 5). In non-navigated sacroiliac screws, the most frequent complication is breach of cortical bone by the screw, which is reported in the literature to occur in up to 35% of cases and which is one of the main reasons for revision surgery (6, 7).

Navigated screw techniques may become especially important given the growing number of elderly patients with reduced bone quality and a higher incidence of low-velocity pelvic fractures (8). For them, the placement of transsacral screws is considered a valid, but more challenging treatment option yet this is not always possible or may be complicated by a dysmorphic sacral configuration (9). In addition, the osseous corridor used for sacroiliac screw placement is noted to be more difficult in the female population, as they have a narrower osseous corridor (10, 11). This is important given that elderly women are more likely to sustain a pelvic fracture (12).

Three-dimensional navigation may allow the surgeon to operate in more challenging osseous conditions, improve overall surgical precision and reduce malposition rates. And indeed, recent studies have reported promising results in small cohorts when using a navigated system for iliosacral screw fixation (13, 14). They suggest improved surgical accuracy compared to non-navigated screw insertion and a decrease of radiation exposure for surgical personnel (15–17). However, studies comparing navigated and non-navigated sacroiliac screw fixation in larger, matched cohorts remain sparse.

It is widely accepted that sacral dysmorphism complicates the procedure of applying of SI or TS screws to the pelvis due to an altered anatomic region such as a steeper angle of the safe osseous corridor (18). Literature reports that especially without 3D-navigation this results in an elevated misplacement rate of the applied screws (16). Nevertheless, there is as yet no broad consensus regarding the precise definition of sacral dysmorphism. Indeed, a number of scores and criteria have been developed in order to facilitate the objective evaluation of this condition (18–21).

This study aims to remedy this. The central hypothesis was that navigated SI-screw placement results in an improved intraoperative precision with fewer rates of malpositioning and less reoperations. In addition, the aim was to identify conditions favoring transsacral screw placement and compare different sacral dysmorphism scores for clinical decision-making.

## Methods

The reporting of this retrospective cohort study was performed according to the STROBE guidelines (STROBE: Strengthening the Reporting of Observational Studies in Epidemiology) (22).

### Setting

The study was conducted in accordance with the Declaration of Helsinki (11) and the Swiss Cantonal Ethics Committee (BASEC No. PB_2016-01888). The study was performed in a Swiss level 1 trauma center (Department of Traumatology, University Hospital Zurich). Data collection and analysis took place from July 2021 to December 2022. All consecutive patients from November 2014 to December 2021 were screened for eligibility retrospectively after the 12-month follow up of which the last one has been performed in December 2022. This timeframe was chosen because an internal generalized informed consent for retrospective analysis of clinical data was introduced in 2014 and allowing a 12-month follow-up period. Follow-up was conducted through regular outpatient consultations with the operating surgeons. All participants gave written consent for the use of retrospective data for scientific research.

### Participants

Each patient who underwent primary percutaneous sacroiliac screw fixation during the recruitment period was screened for eligibility. Identification was performed by manual review of surgical records by two authors and an automated search of the clinical information system.

Inclusion criteria were traumatic injuries/fractures of the posterior pelvic ring with or without concomitant injury and fragility fractures of the sacrum. Other inclusion criteria were age ≥ 16 years, complete data set and signed informed consent.

Exclusion criteria were lack of written consent, active bone infection, pathological fractures, emergency surgeries and intraoperative conversion to open surgery.

### Study groups

Patients were stratified according to the intraoperative imaging modality used. All patients who received 3D-navigated percutaneous sacroiliac screw fixation were defined as the navigated group (group NAV). All navigation was done using the Medtronic StealthStation (S8) (Medtronic Sofamor Danek, Memphis, TN, USA). The control group was defined as patients who underwent the same surgical procedure without 3D navigation, i.e. with either C-arm or O-arm fluoroscopy (group CONV).

### Outcome/baseline variables

The primary outcome parameters are intraoperative accuracy, complications, and reoperation rates. Accuracy was manually measured in postoperative CT scans and clinical data was extracted from the patient history. Parameters included “malpositioning”, “foraminal breach”, “loosening”, “breaking”, “non-union”, “infection”, and reoperation rates. Malpositioning was defined radiologically as any deviation from the safe osseous corridor (contact with the cortical bone) and foraminal breach as a complete violation of the cortical bone on both sides of a foramen (Fig. 2). Each foraminal breach also counts as malpositioning. “Loosening” was defined as presentation of an osteolytic margin, change of positioning in the bone or protrusion of the screw in radiography. The concept of a "safe osseous corridor" for sacroiliac screws pertains to the safe bony channel within the sacrum through which a screw can be placed without causing damage to adjacent neurovascular structures (e.g. sacral nerves or vessels). The establishment of this safe zone is challenging due to the presence of individual anatomical variations, underscoring the importance of preoperative imaging and computer-assisted planning (23). Breakage of screws also was defined radiologically as fragmentation of the osteosynthesis material. Non-union was defined as non-consolidation of the pelvic fracture at least 6 months after surgery, noted on computed tomography. Intraoperative revision/repositioning was defined as any repositioning of the guide wire or screw after first insertion during initial surgery. This information was extracted from the medical record, as well as by manual screening of intraoperative imaging modalities. The number of all intraoperatively performed O-Arm scans was counted, including planning-O-Arm scans done before instrumentation.

The secondary outcome was defined as the correlation between the performed operative procedure (navigation/no navigation), screw selection (SI or TS) and malpositioning rate and a dysmorphic pelvis configuration. Sacral dysmorphism was defined separately by three existing sacral dysmorphism scores (2 × quantitative, 1x. qualitative): (1) A sacral dysmorphism score (DS) above 70 (18), (2) a lateral sacral triangle ratio ≤ 1.5 (BW/BH ratio) (20) and (3) the existence of at least one abnormal morphological pattern of the sacrum as described by Routt et al. (19). There are six abnormal morphological patterns described in the literature with varying specific predictions of an existing sacral dysmorphism (19). For the purpose of statistical evaluation, we determined that the presence of at least one the aforementioned parameters was indicative of a sacral dysmorphism according to this classification. Evaluation was performed in a blinded manner by three authors independently based on the given imaging. Any discrepancies in values or parameters were resolved in a consensus meeting lead by the leading pelvic surgeon.

Other variables of interest included patient characteristics, injury patterns, and fracture classification. Procedural data, such as number of screws used, and which osseous corridors were employed, were collected. A complete overview of these variables is presented in Table 2 and 3.

Fractures were classified by the operating surgeon according to Young and Burgess (24). Insufficiency fractures (IFx) were classified as a separate group (25). Unstable fracture patterns were defined as anterior posterior compression (APC) type II/III, lateral compression (LC) type III, vertical shear (VS), and combined mechanism (CM) according to the Young and Burgess classification (26, 27). At the authors’ centers, unstable pelvis fractures are managed with surgery (28). In stable patterns, surgery is indicated if the patient is unable to mobilize for a prolonged time due to pain.

High and low energy trauma were defined according to the amount of kinetic energy. Falls from standing height were defined as low energy trauma. Higher falls and a greater amount of transmitted kinetic energy (i.e. traffic accidents) were defined as high-energy trauma.

The ISS and NISS were calculated independently by two authors experienced in trauma scoring based on the 2005 AIS edition (29).

### Data extraction

After manual and automated extraction, the data was organized and stored using Microsoft Excel on password-protected in-house computers.

### Data analysis and matching

Continuous data are presented with mean and standard deviation, categorical variables with numbers and percentages. Statistical analysis was performed in R using the “Stat”, “Tableone” “oddsratio” and “irr” packages (30). Propensity score based nearest-neighbor matching was performed in a 1:1 ratio using the “MatchIt” package based on age, gender, and fracture stability (stable/unstable fracture). Figures were created with the “ggplot2” package. Data was visually tested for normality using histograms. Unpaired student T-tests were used for parametric data. Non-parametric data was tested using Wilcoxon–Mann–Whitney tests. Binary outcome categorical data was assessed using a two-sided fisher`s exact test. Non-binary categorical data using chi-squared test. Significance level was set at 0.05.

### Bias and study size

To minimize selection bias and to achieve the maximal available study size within the given study period, all consecutive eligible patients were included. Matching was performed to minimize differences in baseline characteristics that could influence results and to achieve comparability. Data analysis was blinded.

## Results

### Study participants

**Fig. 1:**
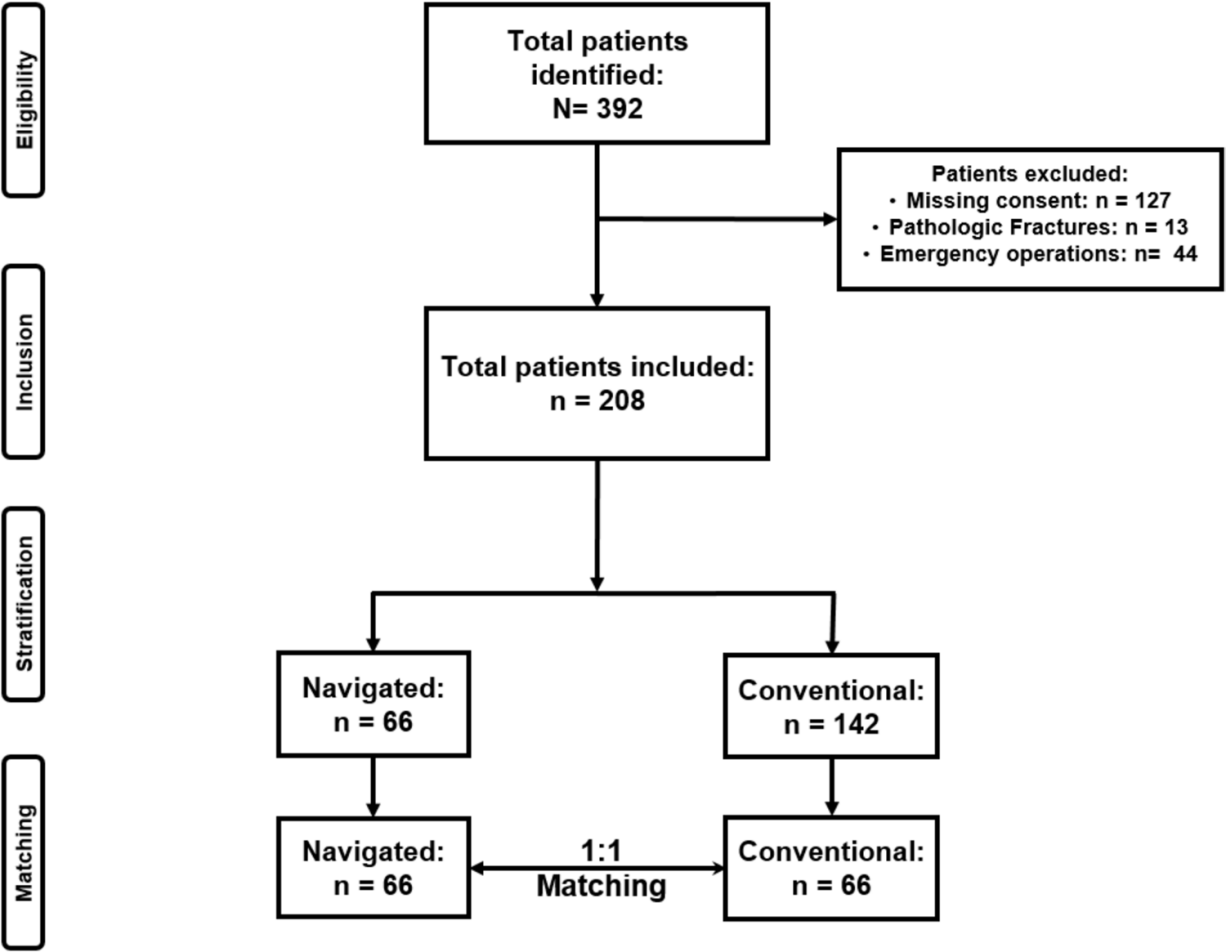
Flowchart of patient inclusion

A total of 392 consecutive patients who received primary percutaneous sacroiliac screw fixation were identified. Of these, 127 patients were excluded due to lack of consent, 13 patients due to pathological fractures and 44 patients due to emergency surgery. 208 patients remained for matching, of which 66 were navigated and 142 were conventionally treated. Those two groups differed in baseline characteristics in terms of trauma kinetics (Table 1). After 1:1 Propensity Score Matching for age, gender and fracture stability, 66 patients remained in each group (Fig. 1). Propensity scores and density plots are presented in the supplementary material (SI) and show high similarities between the matched cohorts.

**Table 1:** Demographics before matching (Significance level p ≤ 0.05)

Pre-matching	Conventional	Navigated	*p*-value
N	142	66	
Age, mean (± SD)	58.85 (± 20.10)	63.02 (± 21.63)	0.189
Age ≥ 60 years, n (%)	73 (51.4)	42 (63.6)	0.103
Female, n (%)	77 (54.2)	40 (60.4)	0.453
Low energy trauma, n (%)	51 (35.9)	35 (53.0)	**0.023**
ISS, median (± SD)	11 (± 9.52)	12 (± 11.29)	0.540
NISS, median (± SD)	17.5 (± 10.52)	13 (± 12.32)	0.365
Unstable Fracture pattern, n (%)	26 (18.3)	19 (28.8)	0.104
Fracture Morphology, n (%)			0.218
APC	10 (7.0)	5 (7.6)	
CM	3 (2.1)	5 (7.6)	
LC	106 (74.6)	41 (62.1)	
VS	7 (4.9)	5 (7.6)	
Insufficiency Fractures	16 (11.3)	10 (15.2)

### Patient demographics

After matching, patients in both groups were similar in terms of age (CONV (mean/SD): 64.26 ± 19.81 years vs. NAV (mean/SD): 63.02 ± 21.63 years; p = 0.731) and contained the same proportion of patients older than 60 years (≥ 60 years) status (CONV: n = 42, 63.6% vs. NAV: n = 42, 63.6%; p = 1). Both groups had more female patients (CONV: n = 39, 59.1% vs. NAV: n = 40, 60.4%; p = 1) and had similar presentation of high and low energy trauma (low energy; CONV: n = 31, 47% vs. NAV: n = 35, 53%; 0.728). Injury severity was comparable in both groups (ISS (median ± SD): CONV: 13 ± 10.11 vs. NAV: 12 ± 11.29; p = 0.941) (NISS (median ± SD): CONV: 17 ± 10.76 vs. NAV: 13 ± 12.32; 0.850). Unstable fracture patterns occurred in 17 cases in the conventional group (25.8%) and in 19 cases (28.8%) in the navigated group (p = 0.845). The distribution of the fracture types is also comparable (p = 0.934) and is shown in Table 2.

**Table 2:** Demographics after matching (Significance level p ≤ 0.05)

Post-matching	Conventional	Navigated	*p*-value
N	66	66	
Age, mean (± SD)	64.26 (± 19.81)	63.02 (± 21.63)	0.731
Age ≥ 60 years, n (%)	42 (63.6)	42 (63.6)	1
Female, n (%)	39 (59.1)	40 (60.4)	1
Low energy trauma, n (%)	31 (47.0)	35 (53.0)	0.728
ISS, median (± SD)	13 (± 10.11)	12 (± 11.29)	0.941
NISS, median (± SD)	17 (± 10.76)	13 (± 12.32)	0.850
Unstable Fracture pattern, n (%)	17 (25.8)	19 (28.8)	0.845
Fracture Morphology, n (%)			0.934
APC	4 (6.1)	5 (7.6)	
CM	3 (4.5)	5 (7.6)	
LC	45 (68.2)	41 (62.1)	
VS	5 (7.6)	5 (7.6)	
Insufficiency Fractures	9 (13.6)	10 (15.2)	

### Surgical treatment

Both groups received stabilization of both S1 and S2 (NAV: n = 49, 74.2%; CONV: n = 40, 60.6%) more often than isolated S1 level (NAV: n = 15, 22.7%; CONV: n = 24, 36.4%) with a non-significant trend to multilevel stabilization in the navigated group (p = 0.629). Isolated stabilization of the S2 level was rare in both groups (NAV + CONV: n = 2, 3%). Most patients of the navigated group underwent transsacral screw placement (n = 46, 69.7%), whereas the conventional group was predominantly treated with a unilateral iliosacral screw placement (n = 39, 59.1%) (p < 0.0001). The total number of levels instrumented was higher in the navigated group (119 levels) than in the conventional group (106 levels). O-Arm scans were commonly performed in the navigated group, with a mean of 2.18 scans (± 0.96 SD) per operation, whereas O-Arm scans in the conventional group were seldom performed (mean/SD 0.14 ± 0.96) (p < 0.0001) (Table 3).

**Table 3:** Screw placement (Significance level p ≤ 0.05)

Matched cohort	Conventional	Navigated	*p*-value
Instrumented level, n (%)			0.629
S1	24 (36.4)	15 (22.7)	
S1 + S2	40 (60.6)	49 (74.2)	
S2	2 (3.0)	2 (3.0)	
Instrumented levels (total amount), n	106	119	
Insertionsite (%)			** < 0.0001**
Bilateral	8 (12.1)	2 (3.0)	
Transsacral	19 (28.8)	46 (69.7)	
Unilateral	39 (59.1)	18 (27.3)	
O-Arm scans, mean (± SD)	0.14 (0.43)	2.18 (0.96)	** < 0.0001**

### Complications

**Fig. 2:**
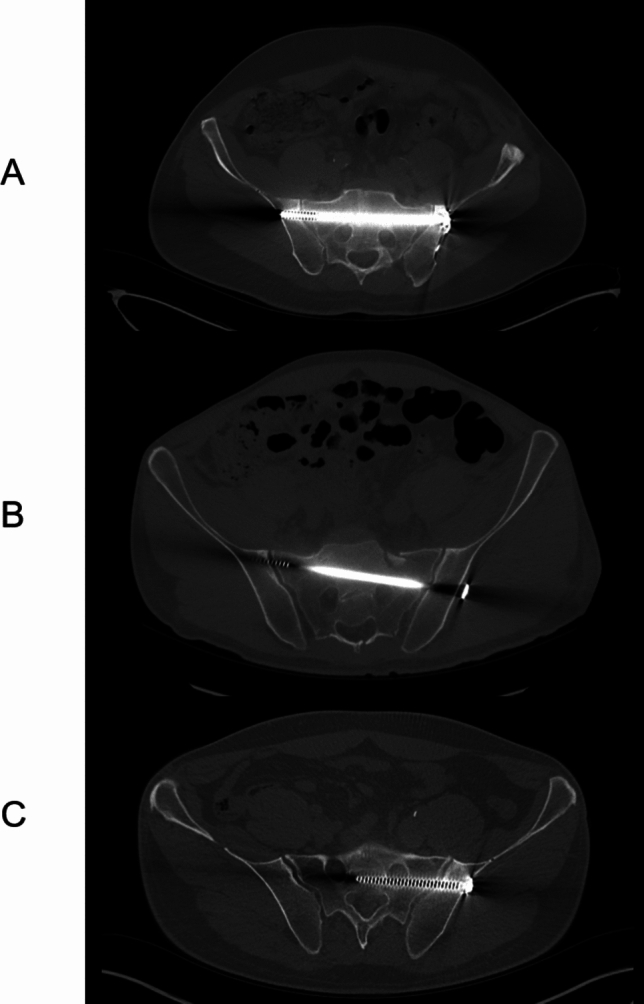
Positioning assessment examples A) Positioning in the safe osseous corridor, B) Malpositioning, C) Foraminal breach

Overall implant complications were not significantly different between the groups (CONV: n = 8, 12.1% vs. NAV: n = 6, 9.1%; p = 1). There was also no difference in complications such as screw loosening (CONV: n = 3, 4.5% vs. NAV: n = 5, 7.6%; p = 0.441), screw breakage (CONV: n = 0, 0% vs. NAV: n = 1, 1.5%; p = 1) and postoperative infection (CONV: n = 2, 3% vs. NAV: n = 0, 0%; p = 0.496). Overall malpositioning and foraminal breaching was also not statistically significant in between the groups. Nevertheless, a clear trend for better intraoperative precision was noted in the NAV Group, in which no malpositioning or foraminal breaching was identified (Malpositioning: CONV: n = 5, 7.6% vs. NAV: n = 0, 0%, p = 0.058; foraminal breach: CONV: n = 3, 4.5% vs. NAV: n = 0, p = 0.244) (Table 4). Examples of correct positioning, malpositioning and foraminal breach are presented in Fig. 2.

**Table 4:** Outcome (Significance level p ≤ 0.05)

Matched cohort	Conventional	Navigated	*p*-value
Implant related complications, n (%)	8 (12.1)	6 (9.1)	1
Loosening, n (%)	3 (4.5)	5 (7.6)	0.441
Malpositioning, n (%)	5 (7.6)	0 (0.0)	0.058
Foraminal breach, n (%)	3 (4.5)	0 (0.0)	0.244
Screw breakage, n (%)	0 (0.0)	1 (1.5)	1
Postoperative infection, n (%)	2 (3.0)	0 (0.0)	0.496
Intraoperative Revision/Repositioning, n (%)	7 (10.6)	10 (15.2)	0.604
Non-union, n (%)	6 (9.1)	2 (3.0)	0.680
Sensoric deficite, n (%)	4 (6.1)	2 (3.0)	0.680
Reoperation, n (%)	13 (19.7)	10 (15.2)	0.816
Reoperation < 3 months, n (%)	1 (1.5)	4 (6.1)	0.365
Reoperation > 3 months, n (%)	12 (18.2)	6 (9.1)	0.299
Reoperation due to complication, n (%)	6 (9.1)	3 (4.5)	0.492
Length of hospital stay (days), mean (± SD)	17.95 (11.80)	14.39 (11.11)	0.076

The intraoperative revision/repositioning (CONV: n = 7, 10.6% vs. NAV: n = 10, 15.2%; p = 0.604) and non-union rate (CONV: n = 6, 9.1% vs. NAV: n = 2, 3%; p = 0.680) in both groups was comparable. There was also no difference in terms of postoperative sensory deficits (CONV: n = 4, 6.1% vs. NAV: n = 2, 3%; p = 0.496. Overall reoperation rates were similar in both groups (CONV: n = 13, 19.7% vs. NAV: n = 10, 15.2%; p = 0.816) as were reoperations due to complications (CONV: n = 6, 9.1% vs. NAV: n = 3, 4.5%; p = 0.492). Reoperations in both groups were comparable. Most were elective implant removals done after fracture healing (> 3 months: CONV: n = 12, 18.2% vs. NAV: n = 6, 9.1%; p = 0.299) (< 3 months: CONV: n = 1, 1.5% vs. NAV: n = 3, 6.1%; p = 0.365). There was no significant difference in the length of hospital stay between the two groups (CONV (mean/SD): 17.95 ± 11.8 days vs. NAV (mean/SD): 14.39 ± 11.11 days; p = 0.076) (Table 4).

The results of the matched cohort analysis indicated that there were no statistically significant differences in the incidence of implant-related complications, loosening, or malpositioning, despite a statistically significant difference of 0% vs. 7.6% in malpositioning between the two groups (p = 0.058). To further assess whether this lack of significance was influenced by limitations in sample size and statistical power, a back-analysis was conducted using the larger unmatched cohort. This approach was undertaken to enhance the sensitivity of detecting clinically meaningful differences, particularly for outcomes with low incidence rates. The objective of utilizing the unmatched cohort was to conduct a more robust assessment of the outcomes, thereby ensuring a more accurate reflection of potential group differences.

In the larger unmatched cohort, malpositioning reached significance with a rate of 7% (n = 10) in the conventional and 0% in the navigated group (p = 0.033). Further outcome parameters did not change significantly (Table 5).

**Table 5:** Outcome in unmatched cohort (Back-analysis) (Significance level p ≤ 0.05)

Unmatched cohort	Conventional	Navigated	p-value
N	142	66	
Implant related complications, n (%)	17 (12.0)	6 (9.1)	0.640
Loosening, n (%)	9 (6.3)	5 (7.6)	0.770
Malpositioning, n (%)	10 (7.0)	0 (0.0)	**0.033**
Foraminal breach, n (%)	6 (4.2)	0 (0.0)	0.180
Screw breakage, n (%)	0 (0)	1 (1.5)	0.317
Postoperative infection, n (%)	2 (1.4)	0 (0.0)	1
Intraoperative Revision/Repositioning, n (%)	11 (7.7)	10 (15.2)	0.077
Non-union, n (%)	10 (7.0)	2 (3.0)	0.346
Sensoric deficite, n (%)	6 (4.2)	2 (3.0)	1
Reoperation, n (%)	34 (23.9)	10 (15.2)	0.201
Reoperation < 3 months, n (%)	6 (4.2)	4 (6.1)	0.729
Reoperation > 3 months, n (%)	28 (19.7)	6 (9.1)	0.069
Reoperation due to complication, n (%)	12 (8.5)	3 (4.5)	0.397
Length of hospital stay (days), mean (± SD)	16.84 (11.11)	14.39 (11.11)	0.142

### Sacral dysmorphism scores

Matched groups were stratified into two groups (dysmorphic and non-dysmorphic) for each of the three defined dysmorphism classifications (Table 4–6). Classification of all three scores was not possible in each patient since this requires different imaging (CT/X-ray) which could not be retrieved/reconstructed in each case, resulting in a varying patient count in between the different scores. The kappa coefficient for the three classifications showed no agreement beyond chance (kappa = 0). The analysis of the three sacral dysmorphism classifications as well as the incidence of complications depending on the existence of dysmorphism by definition is presented in Table 7a-c.

**Table 6:** a) Dysmorphic pelvis classified by a sacral dysmorphism score > 70, B) Dysmorphic pelvis classified by the lateral sacral triangle (BW/BH) ratio ≤ 1.5, C) Dysmorphic pelvis defined by existence of at least one dysmorphic character (**bold** print in the headline indicates dysmorphism according to the score)

**A**	≤ 70	** > 70**	p-value
N	121	9	
Dysmorphic Score mean (± SD)	34.74 (18.26)	79.96 (6.55)	** < 0.0001**
Navigated procedure, n (%)	64 (52.9)	2 (22.2)	**0.150**
Transsacral screw, n (%)	62 (51.2)	1 (11.1)	**0.041**
Malpositioning, n (%)	5 (4.1)	0 (0)	0.611
Foraminal breach, n (%)	3 (2.5)	0 (0)	0.39
Dysmorphic pelvis by BW/BH ratio, n (%)	15 (12.4)	5 (55.6)	**0.008**
Any dysmorphic character, n (%)	79 (65.3)	7 (77.8)	1

**B**	** ≤ 1.5**	> 1.5	p-value
N	20	77	
BW/BH ratio, mean (± SD)	1.30 (0.12)	2.85 (2.18)	** < 0.0001**
Navigated procedure, n (%)	8 (40)	44 (57.1)	0.095
Transsacral screw, n (%)	5 (25)	42 (54.5)	**0.008**
Malpositioning, n (%)	0 (0)	3 (3.9)	1
Foraminal breach, n (%)	0 (0)	2 (2.6)	1
Dysmorphic pelvis by Dysmorphic Score, n (%)	5 (25)	2 (2.6)	**0.008**
Any dysmorphic character, n (%)	20 (100)	54 (70.1)	**0.005**

**C**	0 Characters	** ≥ 1 Character**	p-value
N	24	86	
Navigated procedure, n (%)	11 (45.8)	47 (54.7)	0.319
Transsacral screw, n (%)	13 (54.2)	40 (46.5)	0.665
Malpositioning, n (%)	2 (8.3)	2 (2.3)	0.413
Foraminal breach, n (%)	2 (8.3)	0 (0)	0.092
Dysmorphic pelvis by BW/BH ratio, n (%)	0 (0)	20 (23.3)	**0.005**
Dysmorphic pelvis by Dysmorphic Score, n (%)	0 (0)	7 (8.1)	1

**Table 7:** Comparison of navigated versus conventionally instrumented transsacral screws (Significance level p ≤ 0.05)

Transsacral Screws only (unmatched)	Conventional	Navigated	p-value
n	27	46	
Age, mean (± SD)	72.44 (± 13.61)	69.35 (± 19.37)	0.437
Age ≥ 60 years, n (%)	22 (81.5)	36 (78.3)	1
Female, n (%)	18 (66.6)	34 (73.9)	0.595
Low energy trauma, n (%)	19 (70.4)	30 (65.2)	0.798
ISS, median (± SD)	9 (9.19)	9 (9.12)	0.640
NISS, median (± SD)	9 (9.55)	9 (9.67)	0.744
Unstable Fracture pattern, n (%)	2 (7.4)	10 (21.7)	0.798
Fracture Morphology, n (%)			0.581
APC	1 (3.7)	3 (6.5)	
CM	0 (0)	1 (2.2)	
LC	16 (59.3)	30 (65.2)	
VS	0 (0)	2 (4.3)	
Insufficiency Fractures	10 (37.0)	10 (21.7)	
Instrumented level, n (%)			**0.014**
S1	12 (44.4)	10 (21.7)	
S1 + S2	12 (44.4)	35 (76.1)	
S2	3 (11.1)	1 (2.2)	
Instrumented levels (total amount), n	39	81	
O-Arm scans, mean (± SD)	0.33 (0.68)	2.28 (0.89)	** < 0.001**
Implant related complications, n (%)	3 (11.1)	2 (4.3)	0.352
Loosening, n (%)	2 (7.4)	2 (4.3)	0.623
Malpositioning, n (%)	1 (3.7)	0 (0)	0.370
Foraminal breach, n (%)	1 (3.7)	0 (0)	0.370
Screw breakage, n (%)	0 (0)	0 (0)	1
Postoperative infection, n (%)	0 (0)	0 (0)	1
Intraoperative Revision/Repositioning, n (%)	4 (14.8)	8 (17.4)	1
Non-union, n (%)	2 (7.4)	2 (4.3)	0.623
Sensoric deficite, n (%)	1 (3.7)	1 (2.2)	1
Reoperation, n (%)	3 (11.1)	3 (6.5)	0.664
Reoperation < 3 months, n (%)	0 (0)	2 (4.3)	0.527
Reoperation > 3 months, n (%)	3 (11.1)	1 (2.2)	0.140
Reoperation due to complication, n (%)	2 (7.4)	1 (2.2)	0.551
Length of hospital stay (days), mean (± SD)	16.11 (11.07)	15.15 (12.27)	0.733

### Transsacral screws

**Fig. 3:**
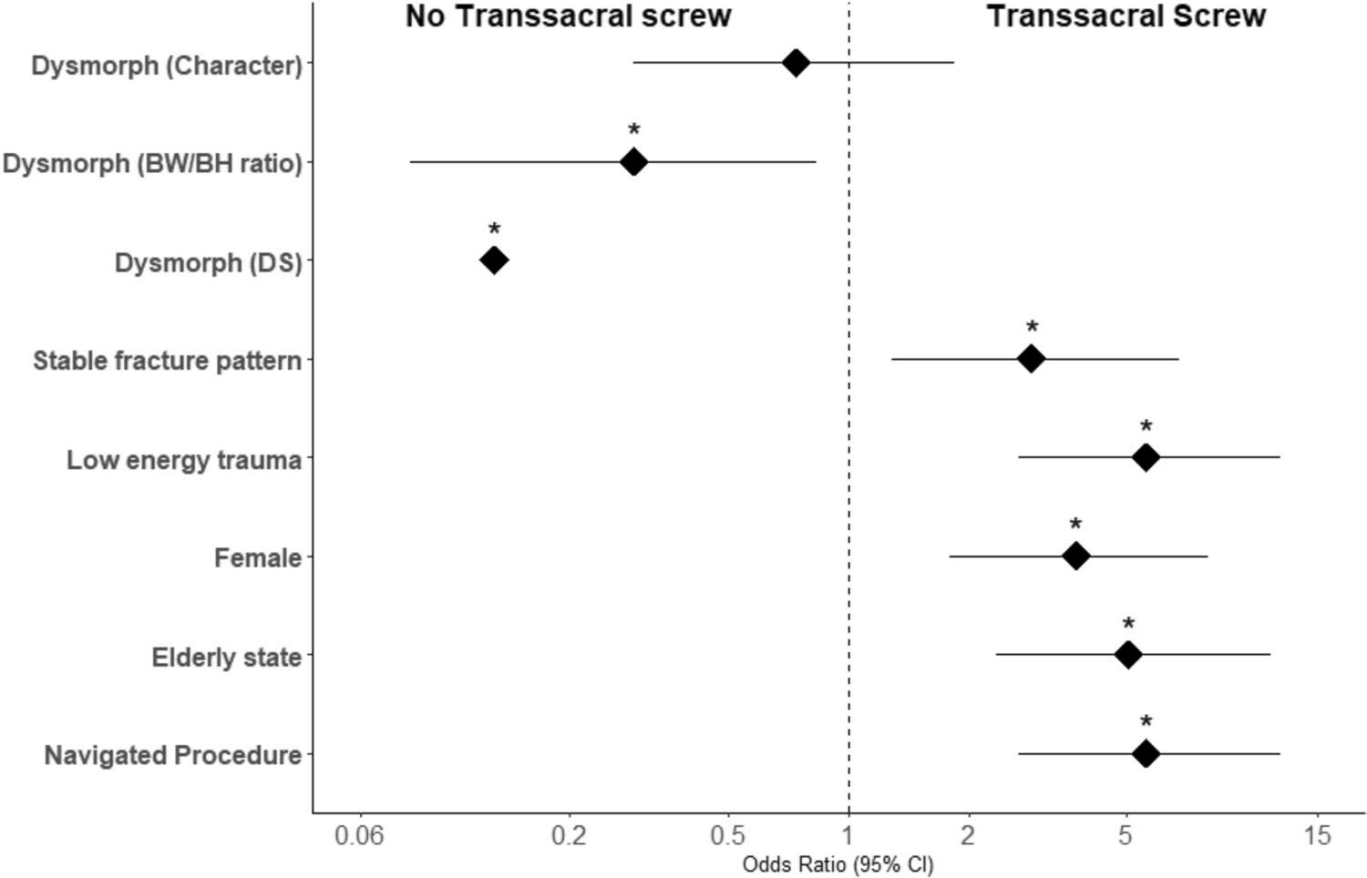
Forest plot with odds ratios for parameters/factors and their association with transsacral screw usage

Transsacral screw placement was significantly more commonly done via a navigated procedure (OR = 5.58, 95%CI: 2.68–12.10, p < 0.0001). Transsacral screws were significantly more commonly done in elderly patients, age ≥ 60 years (OR = 5.03, 95%CI: 2.36–11.50, p < 0.0001), females (OR = 3.72, 95%CI: 1.79–8.01, p = 0.0004), patients with low energy trauma (OR = 5.58, 95%CI: 2.68–12.1, p < 0.0001) and a stable fracture pattern (OR = 2.88, 95%CI:1.29–6.77, p = 0.011) (Fig. 3).

A further (unmatched) subanalysis comparing conventional and navigated insertion of transsacral screws revealed further findings in this regard (Table 6). Overall, 46 patients in the navigated and 27 patients in the conventional group received transsacral screws. Baseline demographics and injury characteristics did not reveal a significant difference. The patients in the navigated group received significantly more multilevel instrumentations on S1 + S2 (CONV: n = 12, 44.4% vs. NAV: n = 35, 76.1; p = 0.014). This resulted in more than twice as much overall instrumented transsacral screws in the navigated group (CONV: n = 39 vs. NAV: n = 81). The main outcomes did not reveal significant differences, potentially influenced by the reduced power of this subanalysis and therefore overall low event rate.

## Discussion

Percutaneous screw fixation is frequently used in patients with posterior pelvic ring injuries, such as dislocations of the sacroiliac joint or fractures of the sacrum (31). This matched-pair analysis of the accuracy and outcome of navigated and conventional posterior pelvic ring screws revealed the following results:

1. Navigated transsacral and unisacral fixation was performed without any malpositioning (0%) or foraminal breach (0%).
2. While there was no statistical difference between the conventional and navigated group in terms of outcome in the matched cohort, significance regarding malpositioning was reached in a back-analysis with increased power indicating improved accuracy with navigation.
3. Transsacral screws were primarily performed in female patients ≥ 60 years of age with low energy trauma and stable fracture types in this study.
4. Sacral dysmorphism scores might be employed to support the decision-making for either transsacral or sacroiliac screw instrumentation.

Similar to other studies, this study demonstrated that the use of navigation for the surgical placement of SI and transsacral screws was accurate and safe. This is the first study to perform a matched-pair analysis and compare patients of the same age, gender and pathology of the pelvic ring injury.

Malpositioning can lead to iatrogenic damage of the nearby neurovascular structures and induce a motoric/functional deficit (32, 33). One way to reduce malpositioning is to use navigation in surgical treatment of posterior pelvic ring injuries, which has also been also demonstrated by Haveman et al. in a recent meta-analysis (34). With use of navigation, other investigators have noted low malposition rates, close to or at 0% (14, 16). Takeba et al. reported zero screw malpositions using the same navigation device as utilized in this study (Medtronic StealthStation) (35). In recent years, a plethora of navigation systems have been employed and assessed in a variety of contexts. In the field of literature, there has been an increasing focus on the description of 3D C-arm systems (for example, Ziehm Vision FD), optical navigation (for example, Brainlab Curve, Stryker Navigation), cone-beam CT-based systems (for example, Medtronic O-Arm + StealthStation), and robotic platforms (for example, TiRobot™, TINAVI). It has been reported that, across all systems, navigation is regarded as superior to instrumentation under fluoroscopy with regard to accuracy and misplacement (15, 16, 34). Therefore, our findings are in line with the recently growing evidence and are able to strengthen them even further.

It has been posited in the extant literature that utilizing a navigational system necessitates a more protracted setup time (35, 36). Therefore, this time-consuming strategy should not be used in emergencies but elective procedures. Conversely, a navigated procedure with a fast and reliable setup has the potential to reduce operating time to a certain extent, particularly in long or complex cases, due to more accurate surgical maneuvers. It is hypothesized that future technical improvements, particularly in terms of the technical setup and software, may prove to be advantageous. Another reported benefit of navigation is the significant reduction of radiation exposure for operating room staff (37).

To evaluate whether the improved precision in a navigated procedure is really induced by the navigation itself or rather by repeated repositioning after intraoperative CT-scans until adequate placement, the number of intraoperative CT scans and intraoperative revisions was evaluated. As expected, the amount of CT-scans was significantly higher in the navigated group with a mean of 2.18 compared to 0.14 scans in the conventional group. This is reasonable since a CT-scan is normally performed right before the instrumentation and after wire positioning and/or screw insertion. Nevertheless, intraoperative repositioning of either the wire and/or the screw showed no significant differences in between the groups. Therefore, this supports the thesis, that the navigation itself plays a major role in the accuracy of screw positioning despite an elevated amount of O-arm scans.

Percutaneous fixation techniques of the posterior pelvic ring necessitate an understanding of safe corridors and optimal imaging to ensure the safe positioning of screws. In particular, the positioning of transsacral screws can be challenging since they pass both sacral alar zones (38).

Studies indicate a high prevalence (14.5%) of dysmorphic pelvic anatomy overall distributed equally between genders. However female patients generally tend to have a narrower corridor at the S1 and S2 levels (39). In our study, depending on the used classification used we report a dysmorphic pelvic anatomy in 9% (DS) and 21% (BW/BH) of the cases whereas 78% of patients present at least one dysmorphic character. The considerable prevalence of dysmorphic pelvices by the Routt et al. classification may be attributed to the varying degrees of predictive value attributed to different characters for sacral dysmorphism (29–97%). It is unsurprising that studies reporting outcomes and complication rates following percutaneous sacral fixation in this patient population document screw misplacement in up to 32% (16). As demonstrated in the relevant literature, the S1 corridor appears to be more significantly impacted than the S2 corridor in cases of asymmetry or narrowing resulting from a dysmorphic pelvic configuration (40). Teo et al. report that in cases of extreme pelvic dysmorphism, even with navigation, it might not be possible to position sacroiliac screws accurately in the S1 corridor. The authors therefore advise considering instrumentation of the lower (S2) corridor instead (40). Consequently, revision surgery and correction are frequently performed after this kind of surgery. In particular, in the fragile geriatric population, these revision surgeries may be unnecessary and may be associated with a negative postoperative course. In our study population, we did not observe a significant difference in between dysmorphic and non-dysmorphic sacral configurations by any classification in terms of malpositioning.

Given the lack of consensus on the definition of sacral dysmorphism, several scoring systems were evaluated in the study. The quantitative scores, namely the sacral dysmorphism score and lateral sacral triangle score, were found to be more effective in predicting the use of transsacral screws than the qualitative evaluation of dysmorphic characters. This is likely due to the fact that the quantitative scores directly evaluate the operational field that the operating surgeon assesses for preoperative planning based on the computed tomography. These scores may be beneficial in preoperative planning, particularly for less experienced surgeons, as they facilitate the objective assessment of transsacral screw instrumentation.

This study showed a significantly higher number of transsacral screws positioned with navigated support. This further highlights the precision of the navigated procedure. Biomechanical studies have confirmed higher stability of transsacral fixation compared to unilateral fixation techniques and recommended their usage especially for osteoporotic fractures (38, 41). At the authors’ centers, as demonstrated by this study, transsacral screw placement is primarily used in the setting of osteoporotic bone. Navigation facilitates this strategy.

## Strengths & limitations

The study’s main strengths are the size of the cohort and the statistical approach. Initial study size of 392 patients was identified and then in a methodological stepwise procedure stratified into the final groups. A computed matching using the most important variables was undertaken, which made the groups highly comparable. Even after matching, an overall population of 132 patients remained. The only remarkable difference noted seemed to be the greater amount of transsacral screw placement in the navigated group. Since transsacral screws span a greater distance in the osseous corridor, small errors may result in greater malposition at the distal part of the screw. However, the navigated group, which contained more transsacral screws, showed no malposition. It must be noted that that the primary outcome of this study concerned intraoperative accuracy. This was determined solely by CT graphic imaging and does not necessarily correlate with clinical symptoms indicating a need for operative revision. While matching provides enhanced comparability of the study groups, it was done at the expense of the power of this study. Not all sacral dysmorphism scores could be calculated for each patient due to partial unavailability or non-assessability of specifically required radiological data. Further clinical outcome parameters did not reach statistical significance in our analysis, but the absence of evidence does not necessarily imply an absence of evidence. As shown in our publications and those of others, navigation primarily improves the accuracy of intraoperative imaging. However, as mentioned earlier, this does not always directly correlate with clinical outcomes. To evaluate the potentially rarer benefits of navigated procedures further, a greater sample size is required, ideally in a prospective or randomized setup.

Due to the retrospective nature of this study, surgeries were performed by different pelvic surgeons whereas some might have personal preferences, whether or whether not to use a navigation system. Generally one might assume that more experienced surgeons would be more comfortable and operate without enhanced orientation/navigation, while young, less experienced surgeons might appreciate the added support of the navigated technique. If this is true, this study seems to demonstrate that navigation does, in fact, provide support for safer screw placement. Additionally, as the evaluated timeline is from 2014 to 2022, there has been casual fluctuation and perhaps a generational shift among the pelvic surgeons performing this procedure. While surgeon-specific variables may always play a role, only pelvic surgeons with over eight years' experience can perform this kind of operation at our institution, and it may be assumed that they are equally skilled. Despite the characteristic limitation of a retrospective study design, the matching procedure for this report improved the comparability of the given groups, without influencing the surgical decision-making.

## Conclusion

Navigated screw fixation of the posterior pelvic ring resulted in optimal accuracy of screw placement in trauma patients and was significantly more accurate than conventional positioning when back-tested in a larger cohort. Patients with poor bone quality might benefit from the placement of transsacral screws, which can be introduced more safely using navigation. Sacral dysmorphism scores might be employed to inform the decision-making process regarding the choice between transsacral and sacroiliac screw instrumentation. It is recommended that future research efforts concentrate on the identification of specific patient cohorts that demonstrate the greatest benefit from navigated procedures in prospective or randomized trials. Additionally, there is a need to evaluate the most recent and upcoming advances in the field of navigation and robot-assisted surgery.

## Conflict of Interests

The authors declare no competing interests.

## Supplementary Information

Below is the link to the electronic supplementary material.

Supplementary file1 (DOCX 34 KB)

## Data Availability

No datasets were generated or analysed during the current study.

## Abbreviations

3D: Three dimensional
AIS: Abbreviated injury scale
APC: Anterior posterior compression
CM: Combined mechanism
CT: Computed tomography
IFx: Insufficiency fracture
ISS: Injury severity score
LC: Lateral compression
NISS: New injury severity score
DS: Sacral dysmorphism score
SI: Sacroiliac
TS: Transsacral
VS: Vertical shear
