# Supplementary material for: Matched pair analysis of the accuracy and outcome of navigated screw fixation of the posterior pelvic ring
Source: Eur J Orthop Surg Traumatol. 2025 Jul 1;35(1):285. doi: 10.1007/s00590-025-04394-2 (PMC12214010; doi:10.1007/s00590-025-04394-2)
Supplement: Supplementary file 1 — Supplementary file1 (DOCX 34 KB) [file 590_2025_4394_MOESM1_ESM.docx]

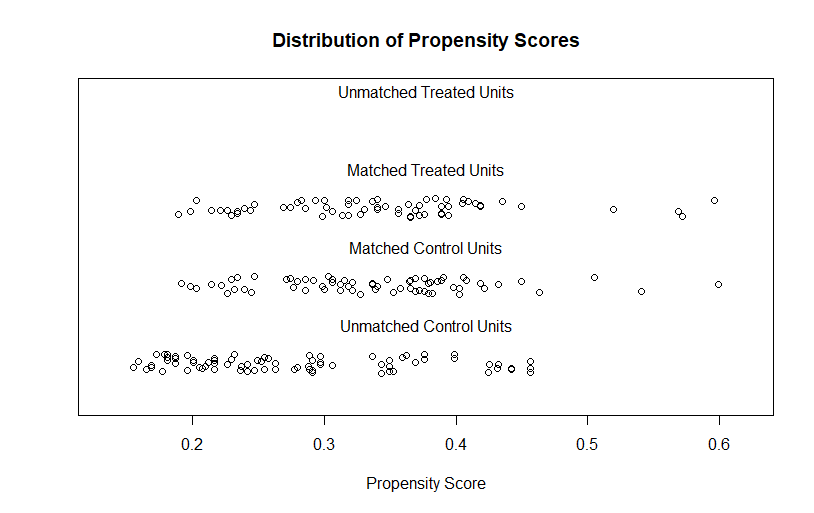


A

B


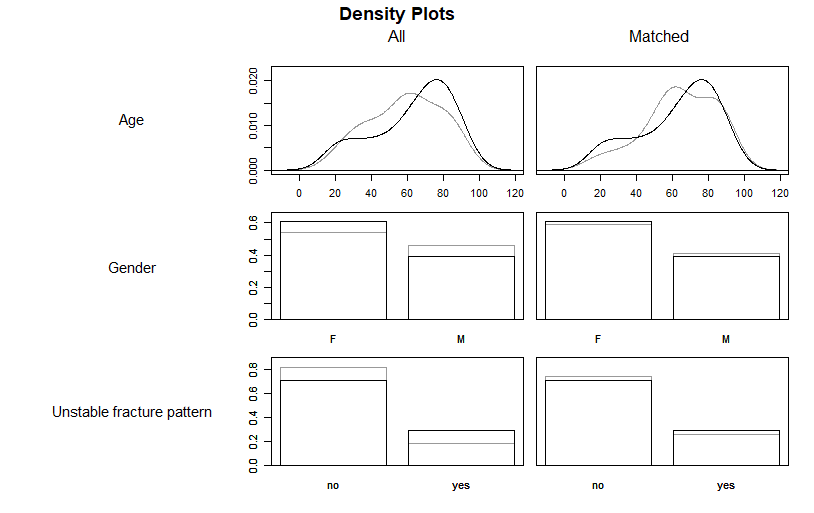


Supplementary 1: A) Distribution of propensity scores after matching, B) Density plots visualizing the distribution of propensity scores for predefined main factors after matching
